# Durability of Concrete Made with Coal Bottom Ash Portland Cements

**DOI:** 10.3390/ma19040773

**Published:** 2026-02-16

**Authors:** Natalia Sanjuán, Silvia Grandes, Miguel Ángel Sanjuán, Pedro López, Aniceto Zaragoza

**Affiliations:** 1 Civil Engineering School, Technical University of Madrid (UPM), C/Professor Aranguren, 3, 28040 Madrid, Spain; 2 Technical Institute of Materials and Construction, Torrejón de Ardoz, 28850 Madrid, Spain; 3 Spanish Institute of Cement and Its Applications (IECA), C/José Abascal, 53, 28003 Madrid, Spain; 4 Oficemen, C/José Abascal, 53, 28003 Madrid, Spain; azaragoza@oficemen.com

**Keywords:** climate change mitigation, IPCC, blended cements, coal bottom ash, building materials, cement, Roadmap 2050, policy

## Abstract

**Highlights:**

**What are the main findings?**

- The standardization of coal bottom ash (CBA) as a cement constituent contributes to climate change mitigation.
- Coal bottom ash (Z) is included as a new constituent in the EU Standardization Request published on 28 July 2025.
- For the first time, the durability of Z-concretes is assessed by the depth of penetration of water under pressure test.
- Clinker factor reduction with newly standardized constituents decreases the near- zero cement cost (~500 €/t).
- CEM II/B-Z and CEM II/C-M concretes meet water penetration requirements for all exposure classes.

**Abstract:**

The Portland cement industry, responsible for approximately 7.4% of global anthropogenic carbon dioxide emissions, must balance rising cement demand with ambitious greenhouse gas reduction targets. In parallel, the rapid accumulation of industrial solid waste highlights the need for effective valorization routes. Reducing the clinker factor remains a powerful measure to mitigate climate impacts in the cement sector. This study evaluates the durability of concretes made with ground coal bottom ash (CBA), a newly standardized Portland cement constituent, using the depth of penetration of water under pressure test (EN 12390-8). The experimental results show that concretes produced with CEM II/B-Z and CEM II/C-M cements meet both average (≤30 mm) and maximum (≤50 mm) water penetration criteria for mass, reinforced, and prestressed concrete across all EN 206-1 exposure classes. Concretes made with CEM VI (S-L) and CEM VI (S-Z) comply for XS1, XS2, XD, XA1, XM, and XF classes. However, for XS3, XA2, and XA3, compliance (≤20 mm and ≤30 mm) is not achieved when using mix design B (300 kg/m^3^, w/c = 0.50). These findings provide robust technical evidence supporting CBA as a viable cement constituent that enhances durability while enabling clinker factor reduction.

## 1. Introduction

Portland cement is the second largest carbon dioxide-emitting industrial sector, generating 7.4% [1] of global anthropogenic emissions, but concrete (made by mixing Portland cement, aggregates, water and chemical admixtures) is the second most used material in the world after water. In addition, demand for Portland cement could increase by up to 45% by 2050 [2], risking a rise in carbon dioxide emissions. Since it is a hard-to-abate sector, carbon capture use and storage (CCUS) techniques are essential to the net-zero or near-zero pathways for Portland cement, but reducing the clinker factor (clinker/cement ratio) could play a supporting role.

Clinker production at high temperatures (>1450 °C) is the highest CO_2_ emission process (around 85%) in cement manufacturing, mainly from burning fossil fuels (pet coke, diesel, etc.) and the limestone calcination process (Table 1). Accordingly, minimizing the clinker factor (clinker/cement ratio) to manufacture low-clinker cement is the most effective procedure to produce low-carbon cements.

Given the high cost of carbon capture utilization and storage (CCUS), currently the first near-zero cement in Europe has an additional cost of above 200% (≈500 €/t in 2026) and it is expected that future low-emission Portland cement will rise by just above 50% [2], making low-carbon cement cheaper and easier to defend. Therefore, the reduction in the clinker factor is a cheaper supporting measure for the mitigation of climate change [3]. Nevertheless, the low availability of traditional Portland cement constituents, primarily coal fly ash or blast-furnace slag, is perceived as the major limitation in implementing this measure for the development of low-carbon cements, because of the phase-out of coal-fired power plants and the change in steel production from blast furnace–basic oxygen furnace (BF-BOF) technology to electric arc furnaces (EAFs) with direct reduced iron (DRI) made with green hydrogen, which is currently available [4]. Consequently, new Portland cement constituents should be explored and standardized in order to encourage widespread usage.

Current Portland cement constituents act in the cementitious system as fillers, promoting clinker phase hydration (limestone), or as pozzolanic and/or hydraulic materials (coal fly ash, ground granulated blast-furnace slag, natural pozzolana, calcined clay, and so on) [5]. When latent hydraulic materials are ground finely, it dramatically increases the surface area and may react with water in the presence of CaO (calcareous coal fly ash and ground granulated blast-furnace slag—high-Ca aluminosilicates) [5]. By contrast, pozzolanic materials, especially high-Si aluminosilicates (siliceous coal fly ash, natural and calcined pozzolana, siliceous coal bottom ash, etc.), can react with calcium hydroxide (Ca(OH)_2_) and water to form C-S-H gel [6].

In the course of this century, many feasibility studies have been launched for the usage of natural and waste resources as new Portland cement constituents. Climate change mitigation and circular economy goals have caused intense research activity in this field. Nevertheless, some potential Portland cement constituents that fulfill all the technical and economic criteria cannot be used since they are not standardized or do not fit existing construction product standards.

Alsubari et al. [7] examined the pozzolanic reaction of palm oil fuel ash (POFA) waste. To reduce the particle size and increase the surface area, it was ground. This process enhanced the pozzolanic reaction. Furthermore, its pozzolanic reactivity was improved by heat treatment.

Kannan et al. [8] confirmed the pozzolanic activity of ceramic waste powder (CWP) using the Frattini test with CWP up to 40%. Heidari and Tavakoli [9] studied blended cements up to 40% of ceramic waste powder (CWP). The compressive strength decreased with the CWP increase at early ages, but it was reduced by increasing the curing time. Lavat et al. examined the pozzolanic properties of ceramic roof tile wastes [10] and they found that the pozzolanic properties of ceramic waste powder (CWP) increased when they were calcined and ground [10]. In addition, they are classified as class F pozzolan based on their chemical composition.

Glass powder (GP) both has an amorphous nature and exhibits a high silica content, which provides a high reactivity in the cementitious system [11]. Such reactivity depends extensively on the particle size [11,12].

Argiz et al. studied ground coal bottom ash to be used in Portland cement [13]. It has suitable physical and chemical properties, reactivity, and compressive strength development, which is very similar to that of coal fly ash [6,13,14]. Therefore, ground coal bottom ash qualifies as a good candidate for Portland cement constituent. In addition, adequate supply volumes can be found [15]. The reduction in the clinker factor with this new standardized pozzolanic addition will help to reduce the current price of the near-zero cement (≈500 €/t in EU 2026).

Recently, Savadogo et al. studied the hydration and physico-mechanical characterization of bottom ash-based cement [16] and Günay et al. assessed the mechanical properties and leaching properties of bottom ash-based mortars [17], concluding that coal bottom ash is a sustainable building material [18]. However, this does not mean coal bottom ash cement can be used in concrete in any EU country. For instance, in Spain, the durability criteria required by the Spanish Structural Code (CodEs) [19] are linked to the “depth of penetration of water under pressure” test [20].

To summarize, palm oil fuel ash (POFA), ceramic waste powder (CWP), glass powder (GP) and ground coal bottom ash are all pozzolanic, i.e., they are siliceous or siliceous/aluminous materials that react with calcium hydroxide produced by Portland cement hydration to form C-S-H gel with binding characteristics. Furthermore, they share a common relationship between the specific surface area and compressive strength. The fineness (specific surface area) of these potential Portland cement constituents and their chemical composition are the key factors in the final Portland cement compressive strength. Generally, a higher specific surface area increases the pozzolanic activity and the early-age compressive strength in concrete. By contrast, the compressive strength can be negatively affected if the water demand is increased.

According to the EU [21,22], the process for delivering Harmonized Technical Specifications (HTSs) lacks coherence. This process was established under the Construction Products Directive (CPD, 89/106/EEC), and continued under the Construction Products Regulation (CPR, 305/2011/EU). In addition, some HTSs do not cover all the basic work requirements (BWR). Accordingly, the European Commission established a method (CPR Acquis) to implement the Construction Products Regulation (CPR 2024/3110), published in December 2024, by systematically reviewing and updating harmonized standards for construction products. The aim of the CPR Acquis is to assess the compliance of the HTSs and legal acts with the current CPR framework [21]. Furthermore, harmonized standards for construction products will include new requirements for environmental sustainability and Digital Product Passports.

The CPR Acquis process started in 2021 for “precast concrete” and “structural metallic products and ancillaries” [23]. On 21st of June 2023 the additional sub-group 6, corresponding to the priority “cement, building limes and other hydraulic binders” (M114 Cement), was launched at the first meeting of the CPR Acquis (E03776/6). The second meeting was on 11 March 2024, the third on 24 June 2024 and the fourth on 24 October 2024.

The scope of the CPR Acquis process was to define the high-level structure of future HTSs in the product area identified by sub-group 6, i.e., scope, essential characteristics, requirements, and so on.

Further to the CPR Acquis process, a new Standardization Request Ad-hoc Group (SRAHG) for cement was created to review the comments on the Standardization Request (SReq) document drawn up by all relevant stakeholders and sent to the European Commission (meetings on 28 March, 5 April, 15 May, 2025). Finally, the SRAHG recommended that a new SReq be accepted by the CEN Technical Board.

During the 39th CEN/TC 51/WG 6 “Definitions and terminology of cement” meeting held on 5 May 2025, it was agreed to initiate a review of the six cement standards linked to this Committee, and during the 40th meeting on 28 August 2025, 24 experts discussed the first prEN 197-1 draft. The deadline for the adoption by the European Standardisation Organisations (ESOs) is 30 June 2027. In particular, 172 essential characteristics were found in the prEN 197-1, and the coal bottom ash and recycled concrete fines are the new cement constituents in this standard [5]. Finally, the EU standardization request (SReq) for cement, lime and other hydraulic binders was published in the *OJEU* on 28 July 2025 [23].

In addition, The European Innovation Council and SMEs Executive Agency (EISMEA) signed a grant agreement with the European Standardization Organization (CEN) in April 2024 to conduct a project called “SuStaCEM” [24], to support Standardisation activities performed by “CEN/TC51 Cement and Building Lime”. This project seeks to incorporate into the standardization process of cements and building limes appropriate materials with a low carbon footprint and new recycled materials to contribute positively to climate change mitigation. Currently, different workstreams are being investigated, i.e., ferrous and non-ferrous slags, by-products other than slags, carbonated minerals, activated materials, biomass products, and other recycled materials. Therefore, the current European way to introduce new cement constituents in the European standards may be realized through the “SuStaCEM” project [24].

This work systematically summarizes the durability results of concrete made with ground coal bottom ash cements assessed by the depth of penetration of water under pressure test. These new cements will be standardized in Europe by 2028. Later, these new standards need to be promoted and enforced for practical applications such as bridges, building, roads, and so on, country by country. This implies national administrative procedures that may be perceived as a regulatory barrier. The world is in a state of climate emergency; therefore, accelerating the shift to low-carbon cements is essential in the context of climate change mitigation.

The objective of this study is to contribute to enforcing the use of these new low-carbon cements for some practical applications by generating new knowledge and overcoming some regulatory barriers.

## 2. Materials and Methods

### 2.1. Raw Materials and Manufacture of Portland Cements 

Portland cement (CEM I) [5], limestone (L), coal siliceous fly ash (V), coal siliceous bottom ash (Z), and blast-furnace slag (S) were prepared and submitted by Cementos Tudela Veguín, S.A., C/Argüelles, 25 33003 Oviedo (Asturias), Spain. Both ashes meet standards EN 197-1 [25] and EN 450-1:2013 [26].

The chemical compositions of the coal bottom ash (Z), coal fly ash (V), limestone (L), blast-furnace slag (S) and cement CEM I are given in Table 2. Most of the elements were analyzed using the molten pearl X-ray fluorescence technique, with a wavelength scattering X-ray spectrometer, Bruker’s S8 Tiger. Loss on Ignition (LOI), Insoluble Residue (IR) and Cl^−^ were measured following the EN 196-2 procedures [27].

### 2.2. Manufacture of Portland Cements 

Six Portland cements were manufactured by mixing and homogenizing the raw materials with the composition (percentage by weight) indicated in Table 3.

The proportions of the cement constituents have been chosen considering the upper limits for CEM II/B-V, CEM II/C-M (S-L) and CEM VI cements in EN 197-1 [5] in order to assess the performance of coal bottom ash (Z), when it is replaced by coal fly ash (V). Likewise, (S-Z) and (Z-S) performances were evaluated for comparison with (S-L) in cements CEM II/C-M and CEM VI.

### 2.3. Concrete Manufacture

The manufacture and curing of four cylindrical test specimens (Ø15 × 30 cm) from each batch and compressive strength testing at 28 days (3 specimens) were performed in accordance with EN 12390-2:2020 [28] and EN 12390-3:2020 [29], respectively. Tap water and two polyfunctional additives, MasterPozzolith 488 N and MasterGlenium SKY 633, provided by Master Builders Solutions Iberia Ctra. de l’Hospitalet, 147, 08940 Cornellà de Llobregat, Barcelona, Spain, were also used (0.29–0.79% by cement weight). Silicious crushed aggregate was utilized with the proportion 52:48 coarse aggregate (4/20 mm):fine aggregate (0/4 mm).

Siliceous aggregates for concrete production were Aggregate Fine (AF) with particle size fraction ranging from 0 mm to 4 mm, medium-graded sand (M), crushed sand (S) and a low-fines content (AF-0/4-M-S-L); and a Coarse Aggregate (Gravel) with particle size fraction ranging from 4 mm to 20 mm, medium-graded gravel (AG-4/20-M-S).

Curing was performed in a climatic chamber at 20 ± 2 °C and relative humidity, RH, ≥95%. Table 4 shows the concrete mix design.

### 2.4. Depth of Penetration of Water Under Pressure Test

The depth of penetration of water under pressure test was performed by following the European standard EN 12390-8 [20]. This water penetration test on coal bottom ash concrete assesses its durability by measuring how deep the water ingress is for a concrete specimen under pressure, typically following EN 12390-8 standard. The sample was exposed to water pressure for a set time (3 days at 0.5 MPa), then split, and the average and maximum depth of the wet zone is measured, and the less deep, the better.

## 3. Results and Discussion

### 3.1. Concrete Compressive Strength

In view of the high price of the first near-zero cement (≈500 €/t in 2026) as result of the high cost of carbon capture and storage (CCS), additional measures to reduce its carbon footprint will be welcome. Some forecasts suggest that full decarbonization with CCS will only double the price of common Portland cement, i.e., only 200 €/t (half the current market price of net-zero cement) [30].

Reducing the clinker factor is often the most effective measure, and a cheaper one, for climate change mitigation [3]. Furthermore, the use of waste streams as potential Portland cement constituents is an interesting approach to removing landfill waste. However, the availability of suitable Portland cement constituents is the major limitation in implementing this measure.

Coal bottom ash is a recent successful case since it has been included in the EU standardization request (SReq) for cement, lime and other hydraulic binders [23]. However, this does not mean coal bottom ash cement can be used in concrete in any EU country. For instance, in Spain, the durability criteria required by the Spanish Structural Code (CodEs) [19] are linked to the “depth of penetration of water under pressure” test [20].

**Figure 1:**
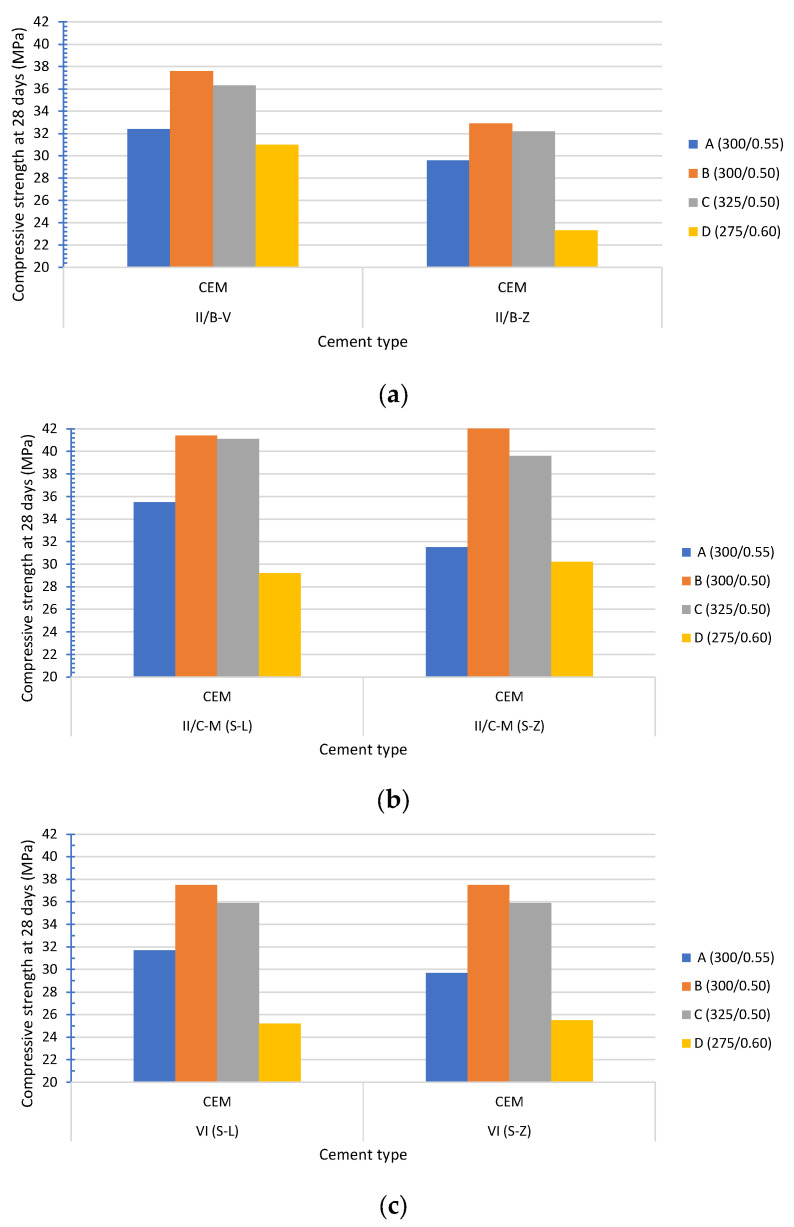
Compressive strength at 28 days (MPa): (**a**) CEM II/B-V and CEM II/B-Z); (**b**) CEM II/C-M (S-L) and CEM II/C-M (S-Z); (**c**) CEM VI (S-L) and CEM VI (S-Z).

Figure 1 shows the compressive strength at 28 days (MPa) for the six types of cement used in the four concrete mix designs. The lowest value was found for CEM II/B-Z in mix D (23.3 MPa), while the highest value was found for CEM II/C-M (S-L) in mix B (41.4 MPa).

X-ray Diffraction (XRD) and scanning electron microscopy (SEM) studies have confirmed the presence of common mineral phases found in coal bottom and fly ash Portland cements, which are quite similar [30,31]. Therefore, differences in the compressive strength can be attributed to their dissimilar specific surface areas, which strongly influence the mechanical strength.

**Figure 2:**
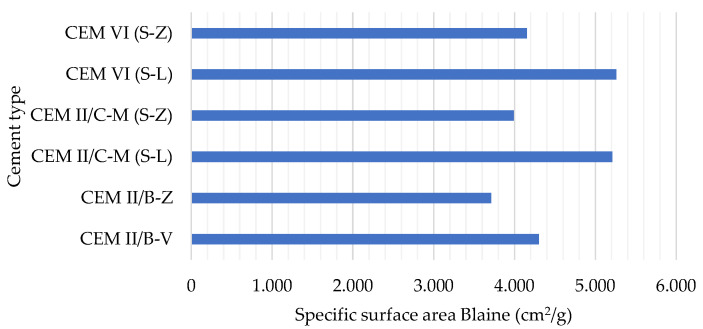
Specific surface area Blaine (cm^2^/g).

Figure 2 shows the specific surface area Blaine measured in the six cements listed in Table 3. Cements made of coal bottom ash (Z) exhibited lower fineness – CEM II/B-Z, CEM II/C-M (S-Z) and CEM VI (S-Z) −. The fineness of coal fly ash cement (4300 cm^2^/g) is significantly higher than that of coal bottom ash cement (3710 cm^2^/g). Given that the matrix of both cements is the same, except for the 35% coal ash (fly or bottom), this difference can only be due to the lower fineness of the coal bottom ash. Accordingly, the compressive strength of CEM II/B-Z is lower than CEM II/B-V (Figure 1a). Surprisingly, this trend is not as pronounced in the case of CEM II/C-M (S-Z) (Figure 1b) and CEM VI (S-Z) (Figure 1c). Moreover, it can be said that the performance is similar in the case of other cements made with coal bottom ash (Z) despite having a lower fineness.

Figure 1a graphically illustrates the differences that exist between both ashes attributed to their different fineness, while Figure 1b compares cements containing limestone (L) or coal bottom ash (Z), as well as blast-furnace slag (S). Coarse limestone powder normally acts as a filler, whereas coal bottom ash presents pozzolanic and filler properties. Greater differences could be expected at a later age.

Figure 1c compares cements with the lowest content of clinker (Table 3). Consequently, the lowest compressive strength data are recorded but follow the same trend as in Figure 1b.

### 3.2. Depth of Penetration of Water Under Pressure

**Figure 3:**
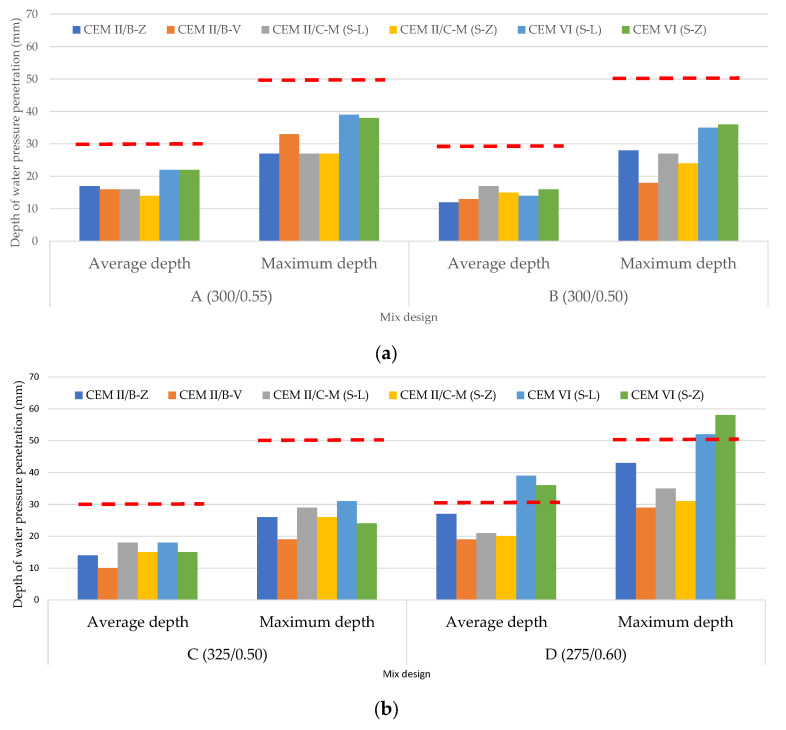
Depth of penetration of water under pressure (mm): average depth and maximum depth: (**a**) mix designs A (300/0.55) and B (300/0.50); (**b**) mix designs C (325/0.50) and D (275/0.60). Red line: upper limit for XS1, XS2, XD1, XD2, XD3, XA1, XM1, XM2, XM3, XF1, XF2, XF3, XF4, XA1 (any type of concrete) and XA2 (mass and reinforced concrete).

The depth of penetration of water under pressure test assesses the permeability and durability of hardened concrete. The deeper the water penetration, the lower the durability. Figure 3 shows the depth of penetration measured in the concretes made according to the mix design presented in Table 4, with the cements listed in Table 3. According to the Spanish Structural Code (CodEs, Chapter 9, Art. 43.3.2 “concrete impermeability”) [19], the durability of structural elements of concrete exposed to highly aggressive environments (XS, XD, XF, XM, or XA, as given in the European standard EN 206 [30]), must be assessed by UNE-EN 12390-8. The criteria for verifying compliance (CodEs, Section 57.3.3 and Table 43.2.2) [19] are set out in Table 5. The set of quality criteria is based on a broad consensus among specialists in the concrete field, national regulators, and experienced users. The classification differs from one class of environmental exposure to another and has two levels of requirement for water depth (average and maximum) (Table 5).

The mix design was selected in order to cover the range of the minimum cement content and maximum water/cement ratio set out for each class of environmental expo-sure in the CodEs [19], in a similar way as in the European standard EN 206 [32].

The minimum cement content and maximum water/cement ratio (w/c) set out in The Spanish is given in Table 6 (CodEs, Table 43.2.1.a) [19]. These parameters are based on a broad consensus among concrete researchers, concrete designers, and national regulators, among other experts. In this case, for each exposure class group and concrete type there is a threshold for cement content and water/cement ratio, as shown in Table 6. First, the composition requirement must be met together with the water penetration requirement as defined in Section 57.3.3 (CodEs), as shown in Table 6 [19]. Later, concretes exposed to the highly aggressive environments listed in Table 5 must be tested.

Performance indicators are widely used by building materials researchers to control the quality of the concrete mix design and/or to estimate the service life of structures built with this concrete. However, they do not provide a holistic assessment of the general performance of concrete mix designs since the concrete durability comprises a large number of durable characteristics, such as corrosion of the steel reinforcement (carbonation and chloride ingress into the concrete cover), freeze–thaw cycles, chemical attack, abrasion, alkali–silica reaction (ASR), and so on. To overcome these limitations, single indicators should be adequately used, converting them into a synthetic indicator which provides a multidimensional assessment of concrete durability performance. Accordingly, there is a growing interest in improving the use of single performance indicators. For instance, electrical resistivity is being used successfully as an effective technique for predicting the durability of reinforced concrete [33]. There is ample evidence that single indicators have room to be employed evaluating concrete durability [34,35,36].

The use of this single indicator provides a tool for assessing the durability of concrete mix designs, which can be considered essential in the regulated concrete sector to develop low-carbon concretes based on low-carbon cements in the context of climate change mitigation. Nevertheless, it does not provide a holistic assessment of the general performance of concrete mix designs since the concrete durability comprises a large number of durable characteristics. This constraint has to be overcome by developing sounder relationships between the durable properties and the single indicators.

The red line in Figure 3 indicates the upper limit criteria for XS1, XS2, XD1, XD2, XD3, XA1, XM1, XM2, XM3, XF1, XF2, XF3, XF4, XA1 (any type of concrete) and XA2 (mass and reinforced concrete) environments according to the CodEs criteria (Table 5) [19]. As can be observed in the bar chart, concretes made with all six cements comply with the CodEs [19] requirement, except for CEM VI concrete made with D (275/0.60). Therefore, the weakest mix design (D) cannot be used to elaborate concrete made with CEM VI when it is going to be utilized to manufacture structural elements in the mentioned environments.

The use of this single indicator provides a tool for ranking mix design concretes according to their respective durability. This information is essential in the regulated concrete sector to develop low-carbon concretes based on low-carbon cements and implement regulatory measures designed to improve climate change mitigation.

The fib Model Code for Concrete Structures underlines that the experimental determination of the water permeability coefficient has not yet been standardized. Nevertheless, the depth of water penetration into concrete can be measured according to EN 12390-8 [20]. Furthermore, it suggests the possibility to convert this value into a water permeability coefficient, but it must be considered as an approximate value only [37]. 

In addition, for mature concrete the water permeability coefficient could also be estimated roughly from the average concrete compressive strength. Since the fib Model Code for Concrete Structures [37] is an international comprehensive code guiding the design and assessment of both new concrete structures, it can be said that this single indicator has certain international relevance. 

The result of the test is the maximum penetration depth rounded to the nearest millimeter. To assess the durability of a particular concrete mix design, the average and maximum water depth of three specimens was considered. Repeatability is generally reliable for dense, low-permeability concrete. Therefore, water penetration depth tests show strong potential for concrete sector adoption. By providing civil engineers with the ability to accurately assess and benchmark new concrete mix designs, this represents a key step toward achieving durable concrete.

Concrete mixes B and C, manufactured with CEM VI (S-L) and CEM VI (S-Z), comply with the average and maximum water penetration depth requirements for mass, reinforced and prestressed concrete structural elements exposed to exposure classes XS1, XS2, XD, XA1, XM, and XF. Furthermore, they also fulfill the test requirements for concrete exposure class XA2 for mass or reinforced concrete. In addition, for concrete exposed to exposure classes XS3 (tidal, splash and spray zones, seawater) and XA3 (highly aggressive chemical environment) for any type of concrete, as well as XA2 (moderately aggressive chemical environment) for prestressed concrete, the results are satisfactory in both CEM VI cements when the C mix design is used (cement content of 325 kg/m^3^ and w/c ratio of 0.50). Nevertheless, the result does not yet entirely meet the criteria with dosage B (cement content of 300 kg/m^3^ and w/c ratio of 0.50). In any case, since the Spanish Structural Code [19] mandates for exposure class XS3 (tidal, splash and spray zones, seawater) the use of sulfate-resistant cement (SRC, SR in EN 197-1) or seawater-resistant cement (MR), CEM VI cements could not have been used in XS3-exposed concrete even if satisfactory results had been obtained in the water pressure penetration depth test.

According to the fib Model Code for Concrete Structures [37], limitations to the concrete porosity are traditionally expressed by the maximum water-to-cement ratio, minimum cement content and moisture curing, following the deemed-to-satisfy approach. By contrast, the use of the depth of penetration of water under pressure test, which is related to the water permeability coefficient, would be technically sounder and politically advisable.

Concrete permeability is a measure of the movement of fluids (gas or liquids) through porous media (concrete). This characteristic highly affects the concrete durability since it is related to the ingress of aggressive chemical species into the concrete. Normally, permeability is estimated from a flow test, which is often more complex than estimating the same concrete’s porosity. Some relationships between porosity and permeability have been proposed in the literature [38].

The reactivity of ground coal bottom ash to be used in Portland cement has been assessed in reference [38], which concluded that ground coal bottom ash has a pozzolanic performance similar to coal fly ash. Therefore, partial or total replacement of coal fly ash by ground coal bottom ash in Portland fly ash cements does not have any significant effect on pozzolanic properties. In addition, some authors have found that both the high specific surface area and the filler effect of fine coal fly ash leads to high compressive strength [6], and durability [39,40,41]. In particular, the fineness of coal fly ash, measured with a 45 μm sieve, has a good correlation with concrete compressive strength [42,43]. A similar improvement in the mechanical properties with the fineness increase has also been reported for other pozzolanic materials [44]. 

## 4. Conclusions

This research work discusses the durability results of concrete made with ground coal bottom ash cements. Durability has been assessed by the depth of penetration of water under pressure test. Performance indicators are necessary in the benchmarking studies used to assess durability of concretes. This paper presents a methodological approach to assess concrete durability, based on the depth of penetration of water under pressure test. The single indicator computed for each concrete mix design is reliable and, therefore, an appropriate tool for facilitating the decision-making process. From a technical and policy perspective, the method and results presented in this study are of key importance since a multidimensional evaluation of the performance of different types of concretes can be achieved by a single indicator, such as the depth of penetration of water under pressure test.

CEM II/B-Z and CEM II/C-M (Z-S) concretes meet CodEs (Spanish Concrete Code) durability criteria for XS1, XS2, XD1-3, XA1, XM1-3, and XF1-4 for all concrete types, and XA2 for mass and reinforced concrete. CEM VI (S-L) and CEM VI (S-Z) concretes comply for XS1, XS2, XD, XA1, XM, and XF (mass, reinforced, prestressed) with mixes B and C, and for XS3 and XA3 (any type) and XA2 (prestressed) with mix C only. However, the Spanish Structural Code mandates for XS3 and XA3 exposure classes the use of sulfate-resistant cement or seawater-resistant cement.

Standardizing coal bottom ash as a cement constituent offers a significant opportunity for climate mitigation by reducing the clinker factor and narrowing the cost gap for near-zero cements (≈500 €/t). Therefore, the standardization of coal bottom ash (CBA) as a cement constituent contributes significantly to climate change mitigation by enabling clinker factor reduction.

These new cements will be standardized in Europe by 2028. Nevertheless, they must be promoted and enforced for practical applications country by country, which might be a regulatory barrier in some cases. Considering the global climate change emergency, the shift to low-carbon cements is essential within climate change mitigation. This study will contribute to enforcing the use of these new low-carbon cements for several practical applications by offering new knowledge and overcoming some regulatory barriers.

The use of this single indicator provides a tool for assessing the durability of concrete mix designs, which can be considered essential in the regulated concrete sector to develop low-carbon concretes based on low-carbon cements in the context of climate change mitigation. Nevertheless, it does not provide a holistic assessment of the general performance of concrete mix designs since the concrete durability comprises a large number of durable characteristics. This constraint must be overcome by developing sounder relationships between the durable properties and the single indicators. Therefore, a considerable amount of research work still remains to be conducted in this field.

Finally, concretes made with CEM II/B-Z or CEM II/C-M meet water penetration requirements for all exposure classes and the methodology proposed in this study can be used for further research programs on assessing the feasibility of new pozzolanic materials as Portland cement constituents, considering their effects not only on compressive strength performance but also on concrete durability. It will help to prevent different types of waste from being deposited in landfills. Moreover, some wastes already accumulated in landfills could be evaluated to be used as potential Portland cement constituents.

## Figures and Tables

**Table 1:** Source of CO_2_ from the preparation of cement (CEM I).

Stage of Portland Cement Production	Process ^1^	Carbon Dioxide Emissions (%)
Clinker raw materials	Quarrying and crushing	0.4
Clinker raw materials	Grinding	1.4
Clinkerization	Fuel burning	38
Clinkerization	Calcination	47
Cement raw materials	Quarrying and crushing	1.2
Cement raw materials	Grinding/blending/packaging	10
Cement (CEM I)	Transportation	4
**Total**		100

^1^ There is an ongoing process of transition to switch from fossil fuels to removable energy sources (H_2_, biomass, etc.), and net-zero electricity use in crushing, grinding and transportation, among other technologies.

**Table 2:** Chemical composition of the coal bottom ash (Z), coal fly ash (V), limestone (L), blast-furnace slag (S) and cement CEM I 42.5 R.

Material	SiO_2_	Al_2_O_3_	Fe_2_O_3_	CaO	MgO	SO_3_	Na_2_O	K_2_O	LOI	IR ^1^	Cl^−^
CEM I 42.5 R	19.5	4.5	4.0	63.7	1.8	3.4	0.1	0.7	2.1	0.4	0.03
S	36.4	12.5	0.5	42.3	6.8	0.1	0.1	0.2	1.0	-	0.01
L	0.5	0.3	0.2	53.9	0.7	0.1	0.1	0.2	43.8	-	0.01
Z	60.80	22.48	12.30	6.1	2.0	0.07	1.35	1.21	0.05	43.85	0.00
V	55.2	21.8	10.2	5.3	1.5	0.1	1.5	2.1	2.3	-	0.03

^1^ Insoluble Residue (IR) was measured by the Na_2_CO_3_ method (EN 196-2) [27].

**Table 3:** Composition of the cements under study.

Cement	Composition (%)				
	Cement (CEM I)	Fly Ash (V)	Bottom Ash (Z)	Blast-Furnace Slag (S)	Limestone (L)
CEM II/B-V	65	35	--	--	--
CEM II/B-Z	65	--	35	--	--
CEM II/C-M (S-L)	50	--	--	25	25
CEM II/C-M (Z-S)	50	--	25	25	--
CEM VI (S-L)	35	--	--	45	20
CEM VI (S-Z)	35	--	20	45	--

**Table 4:** Concrete mix design.

Designation of Dosage	Cement Content (kg/m^3^)	Water/Cement Ratio
A	300	0.55
B	300	0.50
C	325	0.50
D	275	0.60

Concrete mixing was performed in a 100 L ring-pan laboratory concrete mixer.

**Table 5:** Durability criteria for structural concrete exposed to highly aggressive environments based on the depth of penetration of water under pressure (mm) [19].

Class of Environmental Exposure	Maximum Depth (mm)	Average Depth (mm)
XS1, XS2, XD1, XD2, XD3, XA1, XM1, XM2, XM3, XF1, XF2, XF3, XF4, XA1 (any type of concrete)	≤50 mm	≤30 mm
XA2 (mass and reinforced concrete)		
XS3, XA3 (any type of concrete)	≤30 mm	≤20 mm
XA2 (only precast concrete)		

**Table 6:** Maximum water/cement (w/c) ratio and minimum cement content (kg/m^3^) for each exposure class group and concrete type (CodEs, Table 43.2.1.a) [19].

	Concrete Type	Exposure Class Groups										
		XC0	XC1	XC2	XC3	XC4	XS1	XS2	XS3	XD1	XD2	XD3
Maximum Water/Cement (w/c) Ratio	Mass	0.65	-	-	-	-	-	-	-	-	-	-
	Reinforced	0.65	0.60	0.60	0.55	0.55	0.50	0.50	0.45	0.50	0.50	0.50
	Prestressed	0.60	0.60	0.60	0.55	0.55	0.45	0.45	0.45	0.45	0.45	0.45
Minimum Cement Content (kg/m^3^)	Mass	200	-	-	-	-	-	-	-	-	-	-
	Reinforced	250	275	275	300	300	300	325	350	325	325	325
	Prestressed	275	300	300	300	300	300	325	350	325	325	325
	**Concrete type**		**XF1**	**XF2**	**XF3**	**XF4**	**XA1**	**XA2**	**XA3**	**XM1**	**XM2**	**XM3**
Maximum Water/Cement (w/c) Ratio	Mass		0.55	0.50	0.55	0.50	0.50	0.50	0.45	0.50	0.50	0.50
	Reinforced		0.55	0.50	0.55	0.50	0.50	0.50	0.45	0.50	0.50	0.50
	Prestressed		0.45	0.50	0.45	0.50	0.50	0.45	0.45	0.50	0.50	0.50
Minimum Cement Content (kg/m^3^)	Mass		275	300	275	300	275	300	325	300	300	300
	Reinforced		300	325	300	325	325	350	350	325	325	325
	Prestressed		300	325	300	325	325	350	350	325	325	325

## Data Availability

The original contributions presented in this study are included in the article. Further inquiries can be directed to the corresponding author.

## Abbreviations

The following abbreviations are used in this manuscript:

CEM I	Portland cement
L	Limestone
S	Blast-furnace slag
V	Coal siliceous fly ash
Z	Coal siliceous bottom ash

## References

[1] Sanjuán M.Á., Andrade C., Mora P., Zaragoza A. (2020). Carbon Dioxide Uptake by Cement-Based Materials: A Spanish Case Study. Appl. Sci..

[2] World Economic Forum (2022). Net-Zero Industry Tracker; 2022 Edition July 2022.

[3] Sanjuán M.A., Argiz C., Mora P., Zaragoza A. (2020). Carbon Dioxide Uptake in the Roadmap 2050 of the Spanish Cement Industry. Energies.

[4] Hare B., Grant H., Bowen J. (2025). Hard-to-Abate: A Justification for Delay?.

[5] (2026). Cement—Part 1: Common Cement—Performance Assessment and Declaration; CEN/TC 51/WG 6 “Definitions and terminology of cement”; N 674 prEN_197-1 MatureDraft—implemented comments from CEN Quality check—2026-02-10.

[6] Menéndez E., Argiz C., Sanjuán M.Á. (2021). Reactivity of Ground Coal Bottom Ash to Be Used in Portland Cement. J.

[7] Alsubari B., Shafigh P., Ibrahim Z., Jumaat M.Z. (2018). Heat-treated palm oil fuel ash as an effective supplementary cementitious material originating from agriculture waste. Constr. Build. Mater..

[8] Kannan D.M., Aboubakr S.H., EL-Dieb A.S., Reda Taha M.M. (2017). High performance concrete incorporating ceramic waste powder as large partial replacement of Portland cement. Constr. Build. Mater..

[9] Heidari A., Tavakoli D. (2013). A study of the mechanical properties of ground ceramic powder concrete incorporating nano-SiO_2_ particles. Constr. Build. Mater..

[10] Lavat A.E., Trezza M.A., Poggi M. (2009). Characterization of ceramic roof tile wastes as pozzolanic admixture. Waste Manag..

[11] Elaqra H., Rustom R. (2018). Effect of using glass powder as cement replacement on rheological and mechanical properties of cement paste. Constr. Build. Mater..

[12] Du H., Tan K.H. (2014). Waste Glass Powder as Cement Replacement in Concrete. J. Adv. Concr. Technol..

[13] Argiz C., Menéndez E., Sanjuán M.A. (2013). Effect of mixes made of coal bottom ash and fly ash on the mechanical strength and porosity of Portland cement. Mater. Construcc..

[14] Argiz C., Menéndez E., Moragues A., Sanjuán M.A. (2015). Fly ash characteristics of Spanish coal-fired power plants. Afinidad.

[15] Argiz C., Sanjuán M.A., Menéndez E. (2017). Coal Bottom Ash for Portland Cement Production. Adv. Mater. Sci. Eng..

[16] Savadogo N., Traoré Y.B., Nshimiyimana P., Messan A., Hannawi K., Tsobnang F., Agbodjan W.P. (2024). Hydration and physico-mechanical characterization of bottom ash-based cement. Constr. Build. Mater..

[17] Günay G., Cihan M.T., Güneş E. (2024). Evaluation of mechanical properties and leaching tests results of mortars containing waste bottom ash as replacement of cement. J. Mater. Cycles Waste Manag..

[18] Onaizi A.M., Tang W., Amran M., Liu Y., Sajjad U., Alhassan M. (2024). Towards increased adoption of furnace bottom ash as sustainable building materials: Characterization, standardization, and applications. J. Build. Eng..

[19] Spanish Structural Code (CodEs) (2021). Chapter 9 “Durability of Concrete Structures”.

[20] (2019). Testing Hardened Concrete—Part 8: Depth of Penetration of Water Under Pressure.

[21] European Commission Construction Products Regulation Acquis. Responsible Service: Directorate-General for Internal Market, Industry, Entrepreneurship and SMEs. https://single-market-economy.ec.europa.eu/sectors/construction/construction-products-regulation-cpr/acquis_en.

[22] European Commission GROW.DDG1.C.4. Sustainable Industry and Mobility. Circular Economy and Construction. Criteria to be Used for Prioritizing the CPR Technical Acquis Management. Responsible Service: Directorate-General for Internal Market, Industry, Entrepreneurship and SMEs. Document Date: 02/07/2020—Created by—Publication Date: N/a—Last Update: 03/07/2020. https://ec.europa.eu/docsroom/documents/42128.

[23] European Union (EU) (2025). COMMISSION IMPLEMENTING DECISION on a Standardisation Request to the European Committee for Standardisation as Regards Cement, Lime and Other Hydraulic Binders in Support of Regulation (EU) 2024/3110 of the European Parliament and of the Council; Reference C(2025)4828. M/615. 28.07.2025; Responsible Service: Directorate-General for Internal Market, Industry, Entrepreneurship and SMEs.

[24] SuStaCEM (2023). Project Description. New Sustainable Cements and Building Limes. https://www.cencenelec.eu/media/CEN-CENELEC/News/Brief%20News/2024/sustacem_project-description.pdf.

[25] (2011). Cement—Part 1: Composition, Specifications and Conformity Criteria for Common Cements.

[26] (2012). Fly Ash for Concrete—Part 1: Definition, Specifications and Conformity Criteria.

[27] (2015). Methods of testing cement — Part 2: Chemical analysis of cement.

[28] (2019). Testing Hardened Concrete—Part 2: Making and Curing Specimens for Strength Tests.

[29] (2019). Testing Hardened Concrete—Part 3: Compressive Strength of Test Specimens.

[30] Fennell P., Driver J., Bataille C., Davis S.J. (2022). Going net zero for cement and steel. Nature.

[31] Menéndez E., Argiz C., Recino H., Sanjuán M.Á. (2022). Characterization of Mortars Made with Coal Ashes Identified as a Way Forward to Mitigate Climate Change. Crystals.

[32] (2016). Concrete—Specification, Performance, Production and Conformity.

[33] Alonso C., Andrade C., González J.A. (1988). Relation between resistivity and corrosion rate of reinforcements in carbonated mortar made with several cement types. Cem. Concr. Res..

[34] Santos Cortés L.d.C., Zamora Castro S.A., Tejeda del Cueto M.E., Azotla-Cruz L., Lomeli J.S., Velázquez Camilo Ó. (2025). Bulk Electrical Resistivity as an Indicator of the Durability of Sustainable Concrete: Influence of Pozzolanic Admixtures. Appl. Sci..

[35] Torres A.A., Presuel M., Andrade C. (2019). Electrical Resistivity as Durability Index for Concrete Structures. ACI Mater. J..

[36] Andrade C. (2026). Concrete resistivity values for chloride resistance classes. Revista ALCONPAT.

[37] Stuart M. (2024). fib Model Code for Concrete Structures (2020).

[38] Mohammed S.A. (2021). Correlation between permeability and porosity with other properties of concrete. J. Appl. Eng. Sci..

[39] Ferdosian I., Camões A., Ribeiro M. (2017). High-volume fly ash paste for developing ultra-high performance concrete (UHPC). Cienc. Tecnol. Mater..

[40] Payá J., Monzó J., Borrachero M., Peris-Mora E., Amahjour F. (2000). Mechanical treatment of fly ashes: Part IV. Strength development of ground fly ash-cement mortars cured at different temperatures. Cem. Concr. Res..

[41] Aydın S., Karatay Ç., Baradan B. (2010). The effect of grinding process on mechanical properties and alkali–silica reaction resistance of fly ash incorporated cement mortars. Powder Technol..

[42] Lane R.O., Best J.F. (1982). Properties and Use of Fly Ash in Portland Cement Concrete. Concr. Int. Des. Constr..

[43] Ravina D. (1980). Optimized determination of PFA (fly ash) fineness with reference to pozzolanic activity. Cem. Concr. Res..

[44] Sanjuán M., Argiz C., Galvez J., Moragues A. (2015). Effect of silica fume fineness on the improvement of Portland cement strength performance. Constr. Build. Mater..

